# Incidence and impact on outcomes of acute kidney injury after a stroke: a systematic review and meta-analysis

**DOI:** 10.1186/s12882-018-1085-0

**Published:** 2018-10-22

**Authors:** Julia Arnold, Khai Ping Ng, Don Sims, Paramjit Gill, Paul Cockwell, Charles Ferro

**Affiliations:** 1 Department of Nephrology, University Hospitals Birmingham, Birmingham, B15 2WB UK; 2 Department of Stroke, University Hospitals Birmingham, Birmingham, UK; 3 Institute of Applied Health Research, College of Medical and Dental Sciences, University of Birmingham, Birmingham, UK; 4 Warwick Medical School, University of Warwick, Coventry, UK

**Keywords:** Acute kidney injury, Stroke, Cerebrovascular disease, Meta-analysis, Mortality/survival

## Abstract

**Background:**

Patients with chronic kidney disease have worse outcomes after stroke. However, the burden of acute kidney injury after stroke has not been extensively investigated.

**Methods:**

We used MEDLINE and Embase to conduct a systematic review and meta-analysis of published studies that provided data on the risk of AKI and outcomes in adults after ischemic and hemorrhagic stroke. Pooled incidence was examined using the Stuart-Ord method in a DerSimonian-Laird model. Pooled Odds Ratios and 95% confidence intervals were calculated for outcomes using a random effects model. This review was registered with PROSPERO (CRD42017064588).

**Results:**

Eight studies were included, five from the United States, representing 99.9% of included patients. Three studies used established acute kidney injury criteria based on creatinine values to define acute kidney injury and five used International Classification of Diseases coding definitions. Overall pooled incidence was 9.61% (95% confidence interval 8.33–10.98). Incidence for studies using creatinine definitions was 19.51% (95% confidence interval 12.75–27.32%) and for studies using coding definitions 4.63% (95% confidence interval 3.65–5.72%). Heterogeneity was high throughout. Mortality in stroke patients who sustained acute kidney injury was increased (Odds Ratio 2.45; 95% confidence interval 1.47–4.10). Three studies reported risk factors for acute kidney injury. There was sparse information on other outcomes.

**Conclusions:**

Mortality in stroke patients who develop acute kidney injury is significantly increased. However the reported incidence of AKI after stroke varies widely and is underestimated using coding definitions. Larger international studies are required to identify potentially preventable factors to reduce acute kidney injury after stroke and improve outcomes.

**Electronic supplementary material:**

The online version of this article (10.1186/s12882-018-1085-0) contains supplementary material, which is available to authorized users.

## Background

Stroke is the leading cause of neurological disability worldwide with huge social and economic impact [1]. In 2015 there were 6.24 million deaths caused by stroke [2]. Chronic kidney disease (CKD) is associated with an increased risk of stroke [3]. Some of this relates to shared traditional risk factors, for example hypertension, hypercholesterolemia, diabetes mellitus and cigarette smoking [4]. However CKD itself has also been recognized as a risk factor for stroke [5, 6]. In a recent systematic review and meta-analysis comprising 83 studies and 30,392 strokes, Masson et al. demonstrated that stroke risk increased by 7% for every 10mls/min/1.73m^2^ decline in glomerular filtration rate (GFR) [7].

Acute kidney injury (AKI) is a clinical syndrome defined as an abrupt decrease in kidney function resulting in disturbance of fluid, electrolyte and acid-base homeostasis [8]. AKI is a spectrum, ranging from mild, asymptomatic injury to severe injury requiring renal replacement therapy (RRT) [8, 9]. Over the last decade, with the development and wide adoption of international classification systems [8–10], there has been an increasing amount of research into the incidence of AKI and its influence on adverse outcomes in both high and low income countries [11–14].

After a stroke, neurological deficit leading to dysphagia and physical disability, physiological effects including changes in blood pressure and cerebral salt wasting, as well as investigations and treatments, can all potentially contribute to the development of AKI. Furthermore, older, comorbid patients are at greatest risk of AKI [14]. Strategies to prevent AKI in stroke patients could therefore be of great importance. Although the association between CKD and stroke outcomes has been the subject of several systematic reviews and meta-analyses [7, 15], the relationship between AKI and stroke is much less clear. We therefore analyzed the reported rates of AKI incidence after a stroke and the associations between AKI and outcomes after a stroke.

## Methods

Our systematic review was registered with PROSPERO (CRD42017064588) and we adhered to the PRISMA reporting statement [16]. A literature search of MEDLINE was performed from 1946 through to 30 June 2017 using relevant text words and medical subject headings *acute kidney injury, acute kidney failure, acute renal failure, acute renal insufficiency*, combined with *stroke, cerebrovascular disorders and CVA* or *TIA*. Embase was searched from 1974 to 30 June 2017, using the same medical subject headings for AKI as for MEDLINE, combined with *cerebrovascular accident, cerebrovascular disease, cerebrovascular disorder, brain hemorrhage; brain infarction* and *stroke* (for a detailed search strategy see Additional file 1). All searches were limited to human studies with no language restrictions. Authors manually reviewed the reference lists of retrieved articles for additional relevant studies.

### Study selection

Study eligibility was determined using a standardized form (Additional file 2). Two authors, JA and KN, independently screened the list of studies generated by the search, with disagreements resolved by a third author, CF. Titles and abstracts of all studies were screened before obtaining full text versions of relevant studies. To improve generalizability, studies were included if they were a case control or cohort (prospective or retrospective) study and had a sample size greater than 500 adult subjects hospitalized with either an acute ischemic or hemorrhagic stroke [17]. Included studies had a clear statement regarding the definition of acute kidney injury - creatinine values alone were not sufficient. Subarachnoid hemorrhage was not included in this systematic review in view of the different aetiopathophysiology.

### Data collection and analysis

Data was collected using a standardized proforma (Additional file 2) by JA and KN. The following study details were recorded: authors, year of publication, country of publication, type of study, clinical setting, sample size, patient characteristics (age, sex, ethnicity, and comorbidities), definition, type and severity of stroke and definition of AKI. Clinical parameters on admission, including serum creatinine and/or GFR, exposure to radiocontrast media, where specified and number of patients who developed AKI were recorded. Outcomes including mortality, disability, length of stay, re-stroke or cardiac events were also recorded.

Study quality assessment was performed independently by two authors, JA and CF using the Newcastle-Ottawa scale [18]. A maximum of 9 points can be allocated to a particular study based on quality of selection, comparability and study outcome (including follow up). Scores were defined as poor (0–3), fair (4–6) and good (7–9) [17].

Data synthesis, meta-analysis and statistical analysis were performed using Review Manager v5.3.5 software (The Cochrane Collaboration, UK) and StatsDirect v3.0 (StatsDirect Limited, UK). Meta-analysis of proportions was carried out using the Stuart-Ord (inverse double arcsine square root) method in a DerSimonian-Laird (random effects) model. The Odds Ratio (OR) with accompanying 95% confidence intervals (95% CI) were used to report individual and summary effect measures for dichotomous data. Chi squared tests for heterogeneity were performed to examine if the degrees of freedom were greater than the Cochran Q statistic, with α of below 0.05 considered to be statistically significant. In addition, the I^2^ statistic was calculated to provide the estimated percentage of heterogeneity observed. I^2^ values of 25%, 50% and 75% correspond to low, medium and high levels of heterogeneity. Any heterogeneity was further explored. A two-sided *P* value of < 0.05 was considered significant for all analyses.

## Results

### Study characteristics

**Fig. 1:**
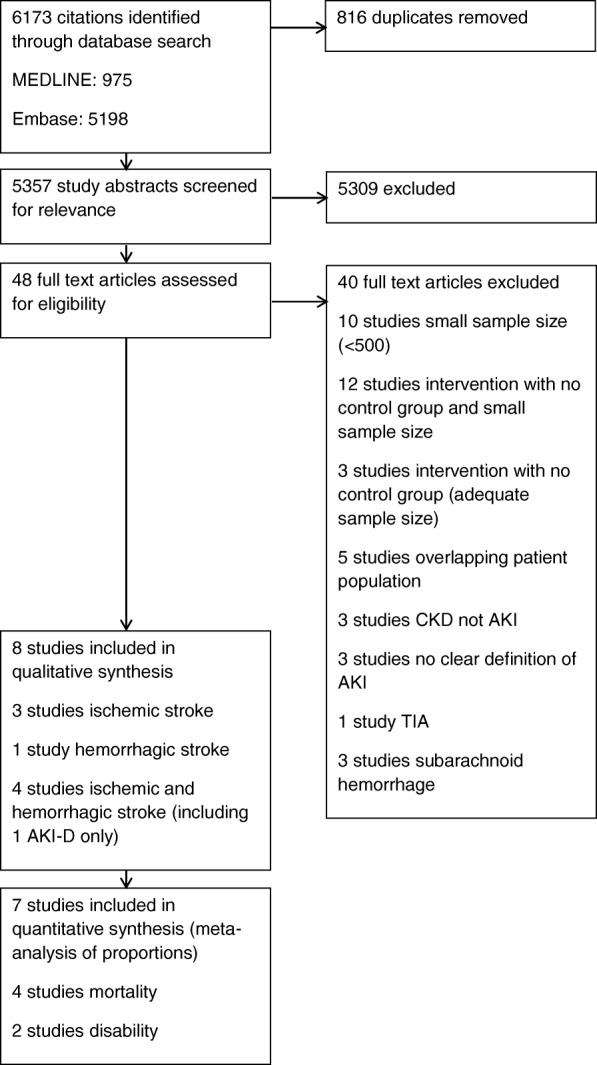
PRISMA flow diagram for literature search and study selection

A total of 6173 potentially relevant citations were identified (Fig. 1), of which 816 were duplicates. A further 5309 articles were excluded after review of title and abstract and an additional 40 excluded after full text review. The characteristics of the eight included studies are displayed in Table 1 [19–26] with the study outcomes summarized in Table 2. All eight studies were published in the English language between 2007 and 2015. Seven studies were considered to be of good quality and one of fair quality. The eight studies provided data on 12,325,652 patients (range 897 to 7,068,334) from four countries (five from the US [20, 22–24, 26], and one each from China [21], Greece [25] and Romania [19]). The US studies overwhelmingly had the largest sample sizes, with a total of 12,319,724 patients representing 99.9% of all included patients. Three of the US studies used Nationwide Inpatient Sample (NIS) data [23, 24, 26]. Although the same database was used, one study included only patients with ischemic stroke [23], one study included only patients with hemorrhagic stroke [24] and a third included only patients who sustained AKI requiring dialysis treatment (AKI-D) [26]. Therefore it was considered appropriate to include only the first two in the meta-analysis [23, 24]. Five out of eight studies used the International Classification of Diseases-9th or 10th Edition (ICD-9/1CD-10) coding to define AKI [21–24] and AKI-D [26]. Only one of the studies reported data on AKI-D in addition to overall AKI incidence [24]. Two studies used the Acute Kidney Injury Network (AKIN) classification [20, 25] and one the Risk/ Injury/ Failure/ Loss/ End-stage (RIFLE) classification [19]. None used urine output criteria. Although there are some differences in the grading of AKI severity between these classifications, both define the absolute incidence of AKI as an increase in serum creatinine > 150%. Stroke was determined using ICD coding in four studies [22–24, 26]. Three studies utilized the World Health Organization (WHO) definition of stroke [27] and extracted clinical data from medical records prospectively [21, 25] or retrospectively [19]. Two studies [21, 25] further subclassified the etiology of ischemic stroke using TOAST criteria [28]. One study utilized stroke registry data as well as clinical records and ICD coding [20].

**Table 1:** Characteristics of the 8 included studies

Author	Type of study/ country	No. of subjects	Age (years) (SD)	Men (%)	Ischemic stroke cases (%)	AKI definition	CKD excluded?	NOS score
Covic et al., 2008 [19]	Observational, retrospective, Romania	1090	66.1 ± 11.5	49.3	932 (85.5%)	Creatinine values; RIFLE	No	7 (3, 2, 2)
Khatri et al., 2014 [20]	Observational, retrospective, United States	1357	64 ± 16	56.0	528 (38.9%)	Creatinine values; AKIN	GFR < 15 ml/min excluded	6 (2, 2, 2)
Lin et al., 2011 [21]	Observational, prospective, China	2683	66.1 ± 13.59 (AF), 63.58 ± 13.64 (no AF)	58.4	2683 (100%)	ICD-10 coding	No	7 (3, 2, 2)
Mohamed et al., 2015 [22]	Observational, retrospective, United States	897	64.4 ± 14.7	44.0	897 (100%)	ICD-9 coding	No	7 (3, 2, 2)
Saeed et al., 2014 [23]	Observational, retrospective, United States (Nationwide Inpatient Sample data)	7,068,334	No AKI 71 ± 31, AKI 74 ± 28	46.1	7,068,334 (100%)	ICD-9 coding	Yes	7 (3, 2, 2)
Saeed et al., 2015 [24]	Observational, retrospective, United States (Nationwide Inpatient Sample data)	614,454	No AKI 69 ± 37, AKI 68 ± 34	52.2	0 (0%) (all cases were hemorrhagic stroke)	ICD-9 coding	Yes	7 (3, 2, 2)
Tsagalis et al., 2008 [25]	Observational, prospective, Greece	2155	70.3 ± 11.9	61.2	1832 (85%)	Creatinine values; AKIN	No	8 (4, 2, 2)
Nadkarni et al., 2015 [26]	Observational, retrospective, United States (Nationwide Inpatient Sample data)	4,634,682	AIS No AKI 73 ± 0.2, AKI 66 ± 0.3 ICH No AKI 69.7 ± 0.13, AKI 65.4 ± 0.21	AIS 50.0 ICH 60.0	3,937,928 (85%)	ICD-9 coding AKI-D only	No	7 (3, 2, 2)

Abbreviations: AIS, acute ischemic stroke; AKI, acute kidney injury; AKI-D, acute kidney injury requiring dialysis; AKIN, Acute Kidney Injury Network; CKD, chronic kidney disease; GFR, estimated glomerular filtration rate; ICD-9/ 10, International Classification of Diseases, 9th/ 10th Revision; ICH, intracranial hemorrhage; mls/min, milliliters per minute; NOS, Newcastle-Ottawa Scale; RIFLE, Risk, Injury, Failure, Loss, End-Stage Renal Disease

**Table 2:** Incidence of AKI, associated factors, measured outcomes and adjustments in the 8 included studies

Study	Follow up	Factors associated with AKI	Crude Mortality in AKI	AKI an independent risk factor for mortality	Disability and AKI	LOS (days) and AKI	Cost and AKI
Covic et al., 2008 [19]	30 days	Age, renal function on admission, IHD, CHF, hemorrhagic stroke	43.1% vs 12.8% (*P* = 0.001)	No	Not reported	Not reported	Not reported
Khatri et al., 2014 [20]	Hospital discharge	Admission creatinine, NIHSS score	AIS: 33% vs 10% (P ≤ 0.001) ICH: 40% vs 30% (P = 0.020)	For AIS only OR 3.08 (95% CI 1.49–6.35, *P* = 0.002) Adjusted for age, sex, race, comorbidities, smoking, CTA, creatinine, NIHSS score	Not reported	Unadjusted AIS: 17.6 vs 8.4 days (*P* ≤ 0.001) ICH: 13.0 vs 8.0 days (*P* ≤ 0.001)	Not reported
Lin et al., 2011 [21]	1 year	Not reported	Not reported	Not reported	Not reported	Not reported	Not reported
Mohamed et al., 2015 [22]	Hospital discharge	Not reported	Not reported	Not reported	Not significant after adjustment	OR 2.63, 95% CI 1.51–4.58 Adjusted for comorbidities, complications, NIHSS score	Not reported
Saeed et al., 2014 [23]	Hospital discharge	Not reported	8.4% vs 2.9% (*P* ≤ 0.001)	OR 2.2 (95% CI 2.0–2.2, *P* ≤ 0.001) Adjusted for age, sex, race, comorbidities, GI bleeding, sepsis, nicotine dependence	OR for moderate/severe disability 1.3 (95% CI 1.3–1.4, P ≤ 0.001) Adjusted as for mortality	Unadjusted 6 vs 4 days (P < 0.0001)	Unadjusted USD 38,613 vs 24,474 (*P* < 0.0001)
Saeed et al., 2015 [24]	Hospital discharge	Not performed	AKI: 28.7% vs 22.4% (*P* ≤ 0.001) AKI-D vs AKI: 50.2% vs 28.4% (*P* ≤ 0.001)	OR 1.5 (95% CI 1.4–1.6, *P* ≤ 0.001) Adjusted for age, sex, race, comorbidities, nicotine dependence, alcohol abuse, hospital bed size, hospital teaching status	OR for moderate/severe disability 1.2 (95% CI 1.1–1.3, *P* ≤ 0.001) Adjusted as for mortality	Unadjusted 12 vs 7 days (P < 0.0001)	Unadjusted USD 104,142 vs 54,315 (*P* < 0.0001)
Tsagalis et al., 2008 [25]	10 years	NIHSS score, CHF, ICH, GFR	30-day mortality 21.8% vs 12.5% (*P* = 0.001) 10-year mortality 75.9% vs 57.7% (*P* = 0.001)	10-year HR 1.24 (95% CI 1.07–1.44, P ≤ 0.01), Adjusted for sex, SBP, hematocrit, comorbidities, brain edema, antihypertensives, statin use	Not reported	Not reported	Not reported
Nadkarni et al., 2015 [26] AKI-D only	Hospital discharge	Not performed	AIS: 31.8% vs 5.6% (*P* ≤ 0.01) ICH: 40.4% vs 28.5% (*P* ≤ 0.01)	AIS: OR 1.30 (95% CI 1.02–1.48, *P* ≤ 0.001) ICH: OR 1.95 (95% CI 1.61–2.36, *P* ≤ 0.01) Adjusted for demographics, hospital characteristics, Charlson comorbidity index and other diagnoses	OR for adverse discharge category AIS: 1.18, 95% CI 1.02–1.37, *P* ≤ 0.01 ICH: 1.74; 95% CI 1.34–2.24, *P* ≤ 0.01 Adjusted as for mortality	Unadjusted AIS: 14.1 vs 3.6 days (*P* ≤ 0.01) ICH: 23.5 vs 5.3 days (*P* ≤ 0.01)	Unadjusted AIS: USD 32,596 vs 8039 (*P* ≤ 0.01) ICH: USD 58,111 vs 11,255 (*P* ≤ 0.01)

Abbreviations: AF, atrial fibrillation; AIS, acute ischemic stroke; AKI, acute kidney injury; AKI-D, acute kidney injury requring dialysis; CHD, coronary heart disease; CHF, congestive heart failure; CT, computerized tomography; CTA, computerized tomography angiography; GFR, glomerular filtration rate; GI, gastrointestinal; ICH, intracranial hemorrhage; IHD, ischemic heart disease; LOS, length of stay; MI, myocardial infarction, mRS, modified Rankin Scale; NIHSS, National Institutes of Health Stroke Scale; OR, Odds Ratio; RIFLE, Risk, Injury, Failure, Loss, End-Stage Renal Disease; SBP, systolic blood pressure; TIA, transient ischemic attack; USD, United States Dollars

Five studies followed patients until discharge from hospital [20, 22–24, 26], one for 30 days [19], one from hospitalization up to one year [21] and one from 30 days up to 10 years [25] (Table 2). Two studies excluded patients with known CKD [23, 24] and two studies excluded patients with end-stage renal disease (ESRD) [20, 26]. Ischemic and hemorrhagic stroke patients were included in four of the studies [19, 20, 25, 26], ischemic stroke alone in three [21–23] and hemorrhagic stroke alone in one study [24] (Table 2).

### Pooled incidence of AKI after stroke

Nadkarni et al. reported the incidence of AKI-D only [26]. This was 0.15% in hospitalizations with acute ischemic stroke and 0.35% in intracranial hemorrhage, with an overall incidence of 0.5%. Saeed et al. reported an overall incidence of AKI-D of 1.7% [24].

**Fig. 2:**
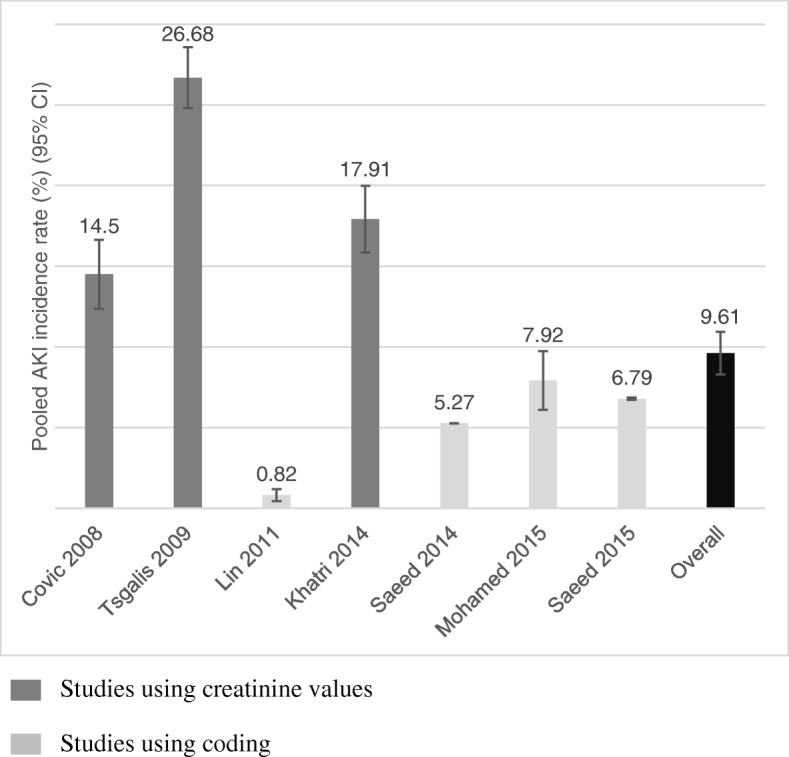
Pooled incidence rates of AKI in all studies listed by year. Data labels are percentages with 95% CI. AKI, acute kidney injury; 95% CI, 95% confidence interval

Using the remaining seven studies, the pooled proportion of AKI as a percentage was 9.61% (95% CI 8.33–10.98) (Fig. 2) with an I^2^ statistic of 99.8% indicating high heterogeneity. Excluding Lin et al. [21], which reported a much lower incidence of AKI than any other study (0.82%), made no difference to the heterogeneity (I^2^ 99.9%).

The pooled incidence of AKI in the studies that utilized ICD coding to define AKI [21–24] was 4.63% (95% CI 3.65–5.72%). Heterogeneity was high (I^2^ 99.9%). Excluding Lin et al. [21], the pooled incidence of AKI increased to 6.46% (95% CI 5.18–7.86%) and heterogeneity remained high (I^2^ 99.9%). Further excluding the study of hemorrhagic stroke hospitalizations [24], the pooled incidence of AKI remained similar (6.42%; 95% CI 4.07–9.27%) with high heterogeneity (I^2^ 90.6%). In comparison, the pooled incidence in studies using creatinine-based AKI definitions [19, 20, 25] was 19.51% (95% CI 12.75–27.32%), again with high heterogeneity (I^2^ 97.4%).

The pooled incidence of AKI in ischemic stroke [19–23, 25] was 9.62% (95% CI 4.20–16.96%; I^2^ statistic 99.5%). Excluding Lin et al. [21], AKI incidence increased to 12.45% (95% CI 4.96–22.70%) and heterogeneity remained high (I^2^ 99.5%). Using studies with a coding definition for AKI, the pooled incidence was 4.05% (95% CI 1.06–8.86%; I^2^ statistic 99.1%). Pooled incidence of AKI in studies utilizing creatinine-based definitions was 17.33% (95% CI 9.42–27.05%) with high heterogeneity (I^2^ 97.6%).

The pooled incidence of AKI in hemorrhagic stroke [19, 20, 24, 25] was 19.17% (95% CI 7.75–34.15%). Heterogeneity was high (I^2^ 99.0%). Excluding Saeed et al. 2015 [24], the only study in this group to use a coding definition for AKI, the pooled incidence increased to 24.50% (95% CI 18.03–31.61%). Heterogeneity decreased but remained high (I^2^ 84.9%).

### Risk factors for AKI after stroke

Factors associated with the development of AKI after multivariate analyses are shown in Table 2. Three studies explored risk factors for the development of AKI after stroke [19, 20, 25]. Older age [19], worse renal function on admission [19, 20, 25], ischemic heart disease [19], heart failure [19, 25] and higher National Institutes of Health Stroke Scale (NIHSS) score on admission [20, 25] were all found to be associated the development of AKI in stroke patients. Use of angiotensin-converting enzyme inhibitors (ACEi) and angiotensin II receptor blockers (ARBs) were only marginally associated with AKI (OR 1.004; 95% CI 0.993–1.058, *P* = 0.057) in one study [19]. One study also tested the association between contrast-enhanced computerized tomography (CT) and AKI and found no relationship [20].

None of the studies presented data on the adjusted rates of AKI associated with cerebral angiography, thrombolysis or any vascular intervention (mechanical thrombectomy, carotid stenting or endarterectomy).

Two studies [19, 25] examined the relationship between stroke type and risk of developing AKI after adjustment for confounders. Covic et al. [19] reported an OR of 2.50 (95% CI 1.42–4.41; *P* = 0.001) in hemorrhagic stroke and Tsagalis et al. [25] an OR of 2.02 (95% CI 1.34–3.04; P = 0.001) with lacunar stroke used as the reference.

### AKI and severity of stroke

Two studies found an association between stroke severity (as determined by NIHSS score) and the development of AKI [20, 25]. In Khatri et al. [20] the adjusted OR per 5 point increase in NIHSS score was 1.13 (95% CI 1.07–1.19; *P* < 0.001). Tsagalis et al. [25] reported an OR of 1.02 (95% CI 1.01–1.03; *P* = 0.020) after adjustment for age, sex, presence of atrial fibrillation (AF), serum glucose, hematocrit and antihypertensive agent use in the first 48 hours of admission.

### AKI and degree of disability

Five studies reported disability post stroke with varying definitions. Two studies [21, 22] recorded degree of disability post stroke, as measured by the modified Rankin Scale (mRS). However data from Lin et al. [21] could not be analyzed with respect to AKI. Mohamed et al. [22] found no association between AKI and degree of disability after multiple adjustments. Two studies [23, 24] used coded discharge destination from NIS data as a surrogate marker for disability. Discharge was categorized as none to minimal disability and any other discharge status (home health care, short-term hospital or other facility including intermediate care and skilled nursing home or death) as moderate to severe disability. Both studies found a higher incidence of moderate to severe disability in patients with AKI after adjustment for multiple confounders (Saeed et al. 2014, OR 1.3 (95% CI 1.3–1.4, *P* < 0.0001); Saeed et al. 2015, OR 1.2 (95% CI 1.1–1.3, P < 0.0001)). A further study [26] utilized an ‘adverse discharge’ category to classify patients as being discharged to a nursing care facility, hospice or long-term care hospital. Here AKI-D was associated with increased odds of adverse discharge (adjusted OR 1.18, 95% CI 1.02–1.37, *P* < 0.01 for ischemic stroke, adjusted OR 1.74; 95% CI, 1.34–2.24; P < 0.01 for ICH) after adjusting for baseline demographics, hospital-level characteristics, Charlson comorbidity index and concurrent diagnoses.

### AKI and length of hospital stay and hospitalization costs

Five studies collected data on length of stay (LOS) [20, 22–24, 26]. All studies reported that AKI was associated with an increased length of stay ranging from 2 to 18 extra days spent in hospital (Table 2). In Mohamed et al. [22], this finding persisted after adjustment for age, NIHSS score, previous stroke and insurance status (no OR given, adjusted *P* < 0.0001).

Three studies analyzed crude inpatient costs using NIS data [23, 24, 26] and all showed AKI was associated with increased inpatients costs ranging from 14,139 to 49,827 US Dollars (Table 2).

### AKI and cardiovascular events

One study [25] examined the relationship between AKI after a stroke and long-term cardiovascular events. The probability of having a composite cardiovascular event during the 10-year period was higher in the AKI group than the non-AKI group (cumulative probability 66.8 (95% CI 56.6–76.9) vs 52.7 (95% CI 48.5–56.1); *P* = 0.001). In a Cox multivariable regression, AKI was an independent predictor of new composite cardiovascular events at 10 years (hazard ratio 1.22; 95% CI 1.01–1.48, *P* < 0.05) after adjustment for hypertension, diabetes, stroke subtypes, brain edema on imaging and hematocrit.

### AKI and post-thrombolytic ICH

Saeed et al. 2014 [23] found that patients with AKI were more likely to suffer a post-thrombolytic ICH (OR 1.4; 95% CI 1.3–1.6, *P* < 0.001) after multiple adjustments including age, sex, race/ ethnicity, hypertension, diabetes, AF, dyslipidemia, congestive heart failure, chronic lung disease, myocardial infarction, gastrointestinal bleeding, sepsis and nicotine dependence.

### AKI and mortality

Six studies [19, 20, 23–26] compared mortality in AKI versus non-AKI groups with all reporting increased mortality in patients who developed AKI. Two studies reported higher crude mortality rates associated with severity of AKI [19, 20].

**Fig. 3:**
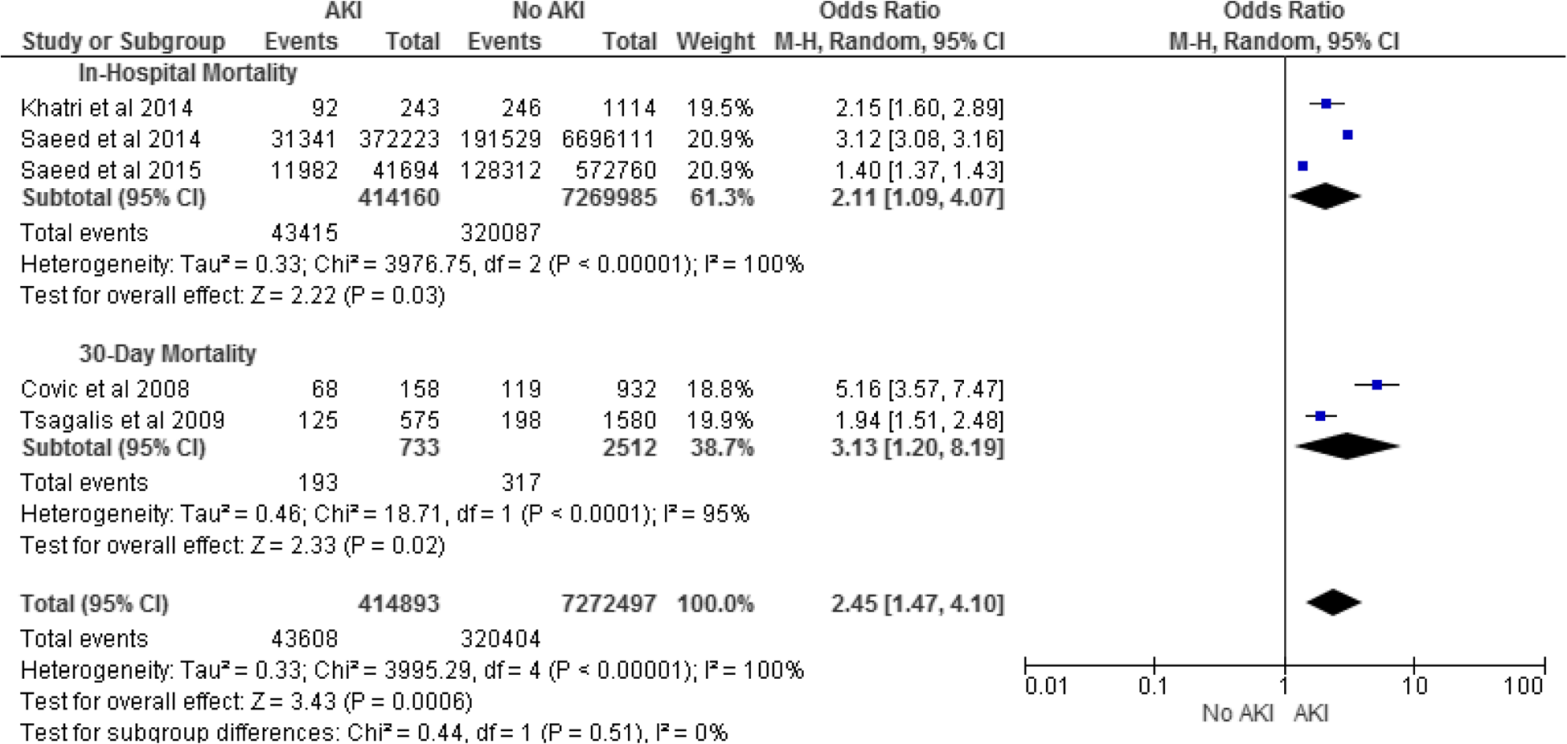
AKI and In-Hospital and 30-Day Mortality for all Stroke. AKI, acute kidney injury; 95% CI, 95% confidence interval

Four studies reported in-hospital mortality [20, 23, 24, 26] and two reported 30-day mortality [19, 25]. The OR for all-cause in-hospital mortality in patients with AKI was 2.11 (95% CI 1.09–4.07) with high heterogeneity (I^2^ 100%; Fig. 3). Excluding Saeed et al. 2015, a study of ICH only, the OR increased to 2.67 (95% CI 1.86–3.83) and heterogeneity decreased but remained high (I^2^ 84%). The OR for all-cause 30-day mortality in patients with AKI was 3.13 (95% CI 1.20–8.19), again with high heterogeneity (I^2^ 95%; Fig. 3).

**Fig. 4:**
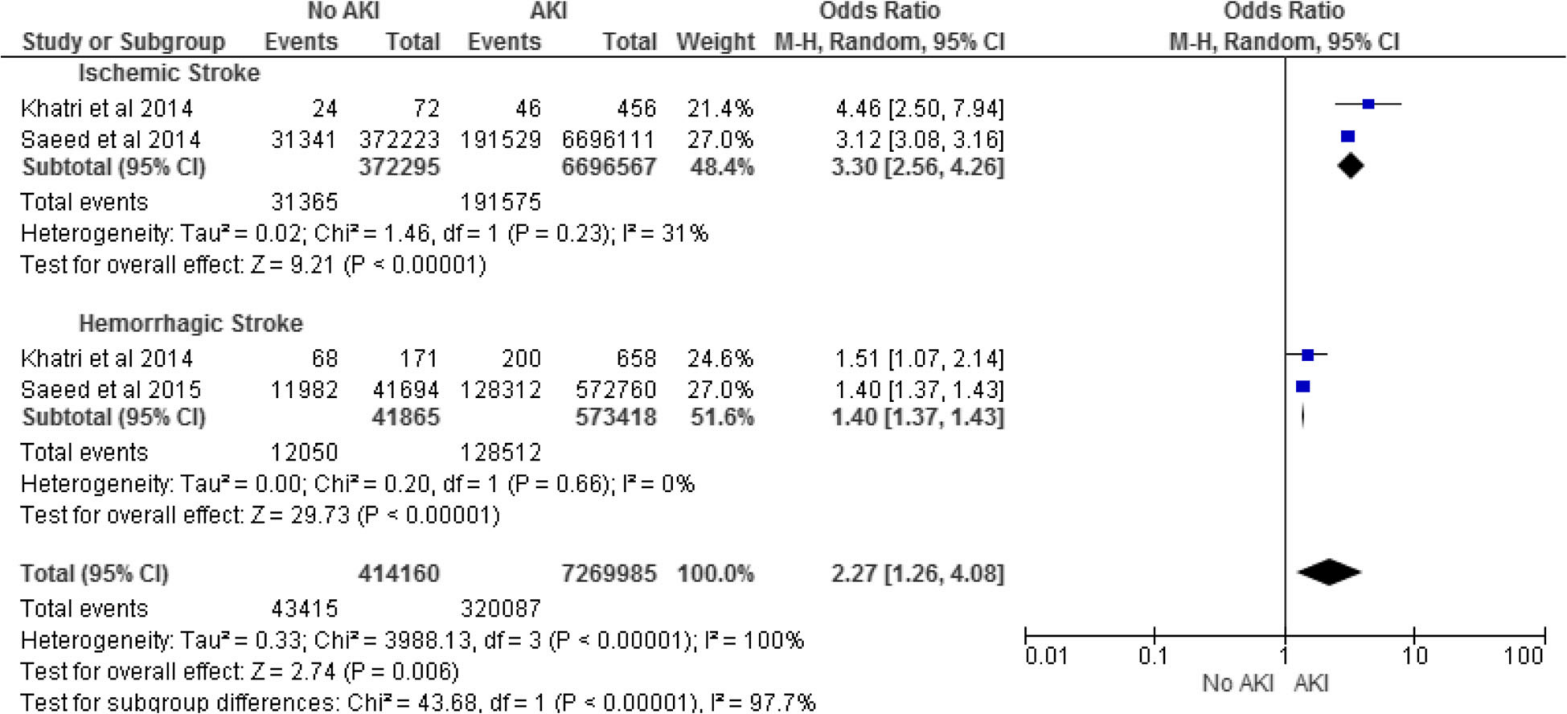
AKI and In-Hospital Mortality for Ischemic and Hemorrhagic Stroke. AKI, acute kidney injury; 95% CI, 95% confidence interval

Two studies [20, 23] provided data on in-hospital mortality after ischemic stroke with a pooled OR of 3.30 (95% CI 2.56–4.26) and low heterogeneity (I^2^ 31%; Fig. 4). Two studies [20, 24] provided data on in-hospital mortality after ICH with a pooled OR of 1.40 (95% CI 1.37–1.43) and low heterogeneity (I^2^ 0%; Fig. 4).

Nadkarni et al. reported an adjusted OR for in-hospital mortality in AKI-D of 1.30 (95% CI 1.12–1.48; *P* < 0.001) in ischemic stroke and 1.95 (95% CI 1.61–2.36; *P* < 0.01) in ICH [26]. Since this study included AKI-D only it was excluded from the meta-analysis. Saeed et al. 2015 reported a higher crude mortality rate in patients with AKI-D than AKI without dialysis (50.2% vs 28.4%, P < 0.001) [24].

Only one study provided long-term mortality data up to 10 years [25], demonstrating higher cumulative mortality in the AKI group at one year (34.6 vs 22.1 in non-AKI group) and 10 years (75.9 vs 57.7; *P* = 0.001). In a Cox proportional hazards model AKI was an independent predictor of 10-year mortality (hazard ratio 1.24; 95% CI 1.07–1.44, P < 0.01) after adjustment for confounders including sex, hypertension, hypercholesterolemia, smoking, systolic blood pressure, brain edema, hematocrit, antihypertensive agent use after the event and ACEi/ARB and statin use on follow-up. The probability of 10-year mortality also increased with severity of AKI.

## Discussion

We have shown that reported rates of AKI after stroke vary widely with a range of 0.82% to 26.68%. Increased severity of stroke is related to the risk of AKI. Furthermore, having an episode of AKI after a stroke is associated with worse disability, increased inpatient mortality, LOS and cost, increased risk of future cardiovascular events and longer-term mortality.

There are marked differences in the reported incidence rates depending on the methodology used to identify patients with AKI. Using coding definitions, the pooled incidence of AKI was 4.63%, compared with 19.51% for definitions based on serum creatinine values. ICD coding is known to underestimate the incidence of AKI [29, 30]. Validation of ICD-9 codes for acute renal failure (ARF, which predates present use of the term AKI), used in Saeed et al. 2014 and 2015 are reported to have a sensitivity of 35.4% and a specificity of 97.7%. Thus the low sensitivity for ARF codes may fail to identify patients with mild AKI that is more likely to go unrecognized and uncoded [30]. We know that severe AKI influences a number of outcomes, at high cost to individual patients, the health service and society [14, 31, 32]. Given that milder categories of AKI are much more common they may be even more important to detect and prevent [11, 14, 33]. Furthermore, apart from Nadkarni et al. [26], only one other study reported data on the incidence of AKI-D and outcomes [24]. Since more severe AKI is related to worse outcomes, this is a significant limitation of our meta-analysis. Only three studies reported on the risk factors associated with AKI after stroke [19, 20, 25] and generally confined themselves to reporting on those already known to be associated with AKI. Only two studies reported that stroke severity at presentation was associated with an increased risk of AKI [20, 25]. Two studies reported that hemorrhagic stroke type was associated with the development of AKI [20, 25]. Interestingly, only one study [20] investigated the relationship between radiological contrast exposure and risk of AKI. None of the studies presented data on the association between thrombolysis, angiographic procedures or vascular intervention in ischemic stroke and AKI. In light of rapid advances in diagnostic scans and interventional treatments for stroke in recent years, including the use of intra-arterial thrombectomy [3], the incidence of AKI in stroke patients may well increase as these interventions become more widespread [3].

Lower baseline renal function was associated with an increased rate of AKI in three of the studies in this systematic review [19, 20, 25]. Patients with CKD are known to have poorer outcomes after a stroke [34–36]. This leads to the question of whether AKI adds further clinical relevance to what is already known about CKD and stroke. Of the studies that excluded patients with CKD [23, 24], AKI was still a clinically significant determinant of both disability and in-hospital mortality after adjustment for multiple confounding factors. Two further studies found AKI was still associated with worse outcomes after adjustment for baseline renal function or CKD category [20, 25]. This supports the theory that AKI is not merely an extension of the CKD spectrum and represents an important standalone factor that predicts worse outcomes in patients with acute stroke.

Our systematic review demonstrates a clear association between AKI and short-term mortality after stroke. This effect persisted after adjustment for CKD in three studies [19, 22, 26]. A further study [25] also found a relationship between AKI and long-term mortality, up to 10 years post stroke, after adjustment for CKD and post-stroke pharmacological treatments. This is consistent with the effect of AKI on short and long-term mortality in the context of other acute illnesses including myocardial infarction, sepsis and major surgery [37–41]. It is therefore, possible that interventions to prevent AKI may improve outcomes after stroke.

Our study has several strengths in that it encompassed several studies, all within the last decade with a large cumulative sample size and a large number of statistical adjustments. However, there are several significant limitations. Firstly, there was disproportionate representation of US data, accounting for 99.9% of the patient sample, which clearly affects generalizability of the estimates. There may also be significant ascertainment bias in view of the US being a high income country with increased availability of blood test monitoring and other diagnostic tests. Secondly, the number of studies included in the systematic review was small and those included in the meta-analysis smaller still. Despite sensitivity analyses, heterogeneity between studies remained substantial potentially suggesting that these studies should not be combined in a meta-analysis. However, we opted to present the data, warts and all, as this both highlights the need for further research in this area but also gives potential future researchers some idea of the numbers needed for recruitment into studies, as well as the rate and range of AKI to be expected using different definitions.

## Conclusions

AKI appears to be a very common complication in hospitalized stroke patients and is associated with increased mortality, disability and healthcare costs. As a potentially preventable condition, further studies are needed in this area to attenuate the effects of AKI, including longer-term morbidity and mortality and the development of CKD. However in the first instance, additional representative studies, ideally using creatinine-based definitions of AKI are required to accurately determine point estimates of AKI post stroke and both short and long-term outcomes.

## Additional files

Additional file 1: Search Strategy. (DOCX 14 kb).

Additional file 2: Data collection proforma. (DOCX 26 kb).

## Abbreviations

ACEi: Angiotensin-converting enzyme inhibitor
AF: Atrial fibrillation
AKI: Acute kidney injury
AKI-D: Acute kidney injury requiring dialysis treatment
AKIN: Acute Kidney Injury Network
ARB: Angiotensin II receptor blocker
ARF: Acute renal failure
CKD: Chronic kidney disease
CT: Computerized tomography
CVA: Cerebrovascular accident
ESRD: End-stage renal disease
GFR: Glomerular filtration rate
ICD-9/10: International Classification of Diseases-9th/10th Edition
ICH: Intracranial hemorrhage
IHD: Ischemic heart disease
mRS: modified Rankin Scale
NIHSS: National Institutes of Health Stroke Scale
NIS: Nationwide Inpatient Sample
OR: Odds ratio
PRISMA: Preferred Reporting Items for Systematic Reviews and Meta-Analyses
PROSPERO: International prospective register of systematic reviews
RIFLE: Risk/ Injury/ Failure/ Loss/ End-stage
RRT: Renal replacement therapy
TIA: Transient ischemic attack
TOAST: Trial of ORG 10172 in Acute Stroke Treatment
WHO: World Health Organization
