# Supplementary material for: Incidence and impact on outcomes of acute kidney injury after a stroke: a systematic review and meta-analysis
Source: BMC Nephrol. 2018 Oct 22;19:283. doi: 10.1186/s12882-018-1085-0 (PMC6196566; doi:10.1186/s12882-018-1085-0)
Supplement: Supplementary file 1 — Search Strategy. (DOCX 14 kb). [file 12882_2018_1085_MOESM1_ESM.docx]

**Additional File 1.** Search strategies in MEDLINE and Embase.

| MEDLINE | EMBASE |
| --- | --- |
| \|  \| 1. acute kidney injury.mp. or exp Acute Kidney Injury/ 2. acute kidney failure.mp. 3. acute renal failure.mp. 4. acute renal insufficiency.mp. 5. 1 or 2 or 3 or 4 6. exp Stroke/ 7. cerebrovascular disorders.mp. 8. cerebrovascular disorders/ or basal ganglia cerebrovascular disease/ or brain ischemia/ or cerebral small vessel diseases/ or "intracranial embolism and thrombosis"/ or intracranial hemorrhages/ or stroke/ 9. stroke.mp. 10. (CVA or TIA).mp. [mp=title, abstract, heading word, drug trade name, original title, device manufacturer, drug manufacturer, device trade name, keyword, floating subheading] 11. 6 or 7 or 8 or 9 or 10 12. 5 and 11 13. Limit 12 to humans \| \| --- \| --- \| | 1. acute kidney failure.mp. or exp acute kidney failure/ 2. acute kidney injury.mp. 3. acute renal failure.mp. 4. acute renal insufficiency.mp. 5. 1 or 2 or 3 or 4 6. exp cerebrovascular accident/ or exp cerebrovascular disease/ 7. cerebrovascular disorder.mp. 8. cerebrovascular accident.mp. 9. cerebrovascular disease.mp. 10. brain hemorrhage.mp. or exp brain hemorrhage/ 11. brain infarction.mp. or exp brain infarction/ 12. stroke.mp. 13. 6 or 7 or 8 or 9 or 10 or 11 or 12 14. 5 and 13 15. Limit 14 to humans |
