# Supplementary material for: Incidence and impact on outcomes of acute kidney injury after a stroke: a systematic review and meta-analysis
Source: BMC Nephrol. 2018 Oct 22;19:283. doi: 10.1186/s12882-018-1085-0 (PMC6196566; doi:10.1186/s12882-018-1085-0)
Supplement: Supplementary file 2 — Data collection proforma. (DOCX 26 kb). [file 12882_2018_1085_MOESM2_ESM.docx]

**Additional File 2.** Data collection proforma.

**Date of screening:**

| **Author(s):** |  |
| --- | --- |
| **Title:** |  |
| **Year of publication:** |  |
| **Country of publication:** |  |
| **Publication type:** | **Journal article/ Other (specify)** |

**Study Eligibility**

|  | **Inclusion Criteria** | **Exclusion Criteria** |
| --- | --- | --- |
| **Type of study** | - **Observational- retrospective/ prospective etc.** |  |
| **Participants** | - **Patients hospitalized with acute stroke** |  |
| **Types of outcome/ measure(s)** | - **AKI incidence** - **Mortality** - **Disability** - **Length of stay** - **Further stroke** - **Further CVS event** - **Factors associated with AKI in stroke patients** - **Other:** |  |
| **Type of intervention(s) (if any)** |  |  |

**Include □ Exclude □**

**Reason(s) for exclusion:**

**………………………………………………………………………………………………………………………………………………………………………………………………………………………………………………………………………………………………………**

| **Study inclusion criteria** |  |
| --- | --- |
| **Study exclusion criteria** |  |
| **Stroke definition e.g. clinical, CT with/ without contrast, first stroke vs any stroke** |  |
| **AKI definition e.g. creatinine values (which ones? e.g. baseline vs imputed values) vs coding, which classification e.g. RIFLE, AKIN**  **Did the study measure urine output?** |  |
| **Setting** | **Source e.g. multicenter, hospital, University teaching hospital** |
| **Study outcome(s) (including duration)** |  |
| **Study intervention(s) (including duration)** |  |
| **Study control (including duration) (if applicable)** |  |
| **Duration of follow up** |  |
| **Compliance/ Loss to f/u (if applicable)** |  |
| **Matching of intervention(s) (if applicable)** |  |
| **Similarity between groups (if applicable)** | **e.g. numbers, dropouts, age, sex** |
| **Statistical analysis** |  |
| **Request for further information**  **Clarification of methods**  **Clarification of results**  **Funding source** |  |

**Results**

| 1. **Participants (entire group) n=** | | | | | | | | |
| --- | --- | --- | --- | --- | --- | --- | --- | --- |
| **Age:** | **Mean (SD)** | | | | **Median (range)** | | |  |
| **Ethnicity:** | **Caucasian** | | **Afro-Caribbean** | **South Asian** | | **Chinese** | | **Other** |
| **Comorbidities** | | | | | | | | |
| **Ischemic heart disease:** | |  | | | | | | |
| **Congestive heart failure:** | |  | | | | | | |
| **Chronic kidney disease (with stages, if given):** | | **1** | | **2** | | | **3a** | |
|  |  | **3b** | | **4** | | | **5 or 5D** | |
| **Diabetes mellitus:** | |  | | | | | | |
| **Hypertension:** | |  | | | | | | |
| **Atrial fibrillation (or other CV embolic condition):** | |  | | | | | | |
| **Previous stroke/ TIA:** | |  | | | | | | |
| **ACEi or ARB** | |  | | | | | | |
| **Stroke type:** | | **Ischemic**  **(Territory if given e.g. total/ partial anterior circulation, posterior, lacunar)** | | | **Hemorrhagic** | | | **SAH/ other** |
| **Clinical parameters** | | | | | | | | |
| **Serum creatinine (including units, mg/dL or umol/L):** | | | | |  | | | |
| **eGFR (if given)** | | | | |  | | | |
| **Acute kidney injury** | | | | |  | | | |
| **Outcomes** | | | | | | | | |
| **Short-term mortality (e.g. 30-day or in-hospital mortality)** | | | | |  | | | |
| **Intermediate mortality (e.g. 3 months)** | | | | |  | | | |
| **Long-term mortality (e.g. 1 year)** | | | | |  | | | |
| **Re-stroke or other cardiac event (if given)** | | | | |  | | | |
| **Disability (and scale of measurement e.g. modified Rankin Scale)** | | | | |  | | | |
| **Length of stay** | | | | |  | | | |
| **Risk factors for AKI identified in the study e.g. age, presence of comorbidities** | | | | |  | | | |
| 1. **Participants (subgrouped e.g. with or without AKI) n=**   **Please specify ______________________________________________________________** | | | | | | | | |
| **Age:** | **Mean (SD)** | | | | **Median (range)** | | |  |
| **Ethnicity:** | **Caucasian** | | **Afro-Caribbean** | **South Asian** | | **Chinese** | | **Other** |
| **Comorbidities** | | | | | | | | |
| **Ischemic heart disease:** | |  | | | | | | |
| **Congestive heart failure:** | |  | | | | | | |
| **Chronic kidney disease (with stages, if given):** | | **1** | | **2** | | | **3a** | |
|  |  | **3b** | | **4** | | | **5 or 5D** | |
| **Diabetes mellitus:** | |  | | | | | | |
| **Hypertension:** | |  | | | | | | |
| **Atrial fibrillation (or other CV embolic condition):** | |  | | | | | | |
| **Previous stroke/ TIA:** | |  | | | | | | |
| **ACEi or ARB** | |  | | | | | | |
| **Stroke type:** | | **Ischemic**  **(Territory if given e.g. total/ partial anterior circulation, posterior, lacunar)** | | | **Hemorrhagic** | | | **SAH/ other** |
| **Clinical parameters** | | | | | | | | |
| **Serum creatinine (including units, mg/dL or umol/L):** | | | | |  | | | |
| **eGFR (if given)** | | | | |  | | | |
| **Outcomes** | | | | | | | | |
| **Short-term mortality (e.g. 30-day or in-hospital mortality)** | | | | |  | | | |
| **Intermediate mortality (e.g. 3 months)** | | | | |  | | | |
| **Long-term mortality (e.g. 1 year)** | | | | |  | | | |
| **Re-stroke or other cardiac event (if given)** | | | | |  | | | |
| **Disability (and scale of measurement e.g. modified Rankin Scale)** | | | | |  | | | |
| **Length of stay** | | | | |  | | | |

| 1. **Participants (subgrouped e.g. with or without AKI) n=**   **Please specify ______________________________________________________________** | | | | | | | | |
| --- | --- | --- | --- | --- | --- | --- | --- | --- |
| **Age:** | **Mean (SD)** | | | | **Median (range)** | | |  |
| **Ethnicity:** | **Caucasian** | | **Afro-Caribbean** | **South Asian** | | **Chinese** | | **Other** |
| **Comorbidities** | | | | | | | | |
| **Ischemic heart disease:** | |  | | | | | | |
| **Congestive heart failure:** | |  | | | | | | |
| **Chronic kidney disease (with stages, if given):** | | **1** | | **2** | | | **3a** | |
|  |  | **3b** | | **4** | | | **5 or 5D** | |
| **Diabetes mellitus:** | |  | | | | | | |
| **Hypertension:** | |  | | | | | | |
| **Atrial fibrillation (or other CV embolic condition):** | |  | | | | | | |
| **Previous stroke/ TIA:** | |  | | | | | | |
| **ACEi or ARB** | |  | | | | | | |
| **Stroke type:** | | **Ischemic**  **(Territory if given e.g. total/ partial anterior circulation, posterior, lacunar)** | | | **Hemorrhagic** | | | **SAH/ other** |
| **Clinical parameters** | | | | | | | | |
| **Serum creatinine (including units, mg/dL or umol/L):** | | | | |  | | | |
| **eGFR (if given)** | | | | |  | | | |
| **Outcomes** | | | | | | | | |
| **Short-term mortality (e.g. 30-day or in-hospital mortality)** | | | | |  | | | |
| **Intermediate mortality (e.g. 3 months)** | | | | |  | | | |
| **Long-term mortality (e.g. 1 year)** | | | | |  | | | |
| **Re-stroke or other cardiac event (if given)** | | | | |  | | | |
| **Disability (and scale of measurement e.g. modified Rankin Scale)** | | | | |  | | | |
| **Length of stay** | | | | |  | | | |

**Loss to follow up/ missing data/ other comments:**

**……………………………………………………………………………………………………………………………………………………………………………………………………………………………………………………………………………………………………………………………………………………………………………………………………………………………………………………………………………………………………………………………………………………………………………………………………………….**

**Reasons for exclusion/ loss to follow up:**

**………………………………………………………………………………………………………………………………………………………………………………………………………………………………………………………………………………………………………………………………………………………………………………………………………………………………………………………………………………………………………………………………………………………………………………………………………………**
